# Supplementary material for: ‘Making the System Work’: A Multi-Site Qualitative Study of Dietitians’ Use of iEMR to Support Nutrition Care Transitions for Older Adults with Malnutrition
Source: Healthcare (Basel). 2025 Sep 5;13(17):2227. doi: 10.3390/healthcare13172227 (PMC12428660; doi:10.3390/healthcare13172227)
Supplement: Supplementary file 1 [file healthcare-13-02227-s001.zip › Document S1_Interview Guide.pdf]

Document S1: Semi-Structured Interview Guide

Interview Purpose and Structure

This semi-structured interview guide was designed to explore clinical dietitians' experiences using the integrated electronic medical record (iEMR) system for discharge planning and coordination of post-discharge nutrition care for older adults with malnutrition transitioning from hospital to home.

Interview Introduction

"The purpose of this interview is to understand your experiences with and views around using iEMR to support the delivery of nutrition care for malnourished older adults transitioning from hospital to home. We are specifically interested in your views on discharge planning through to the coordination of post-discharge nutrition care. We are interested in your opinions; there are no right or wrong answers. As outlined in the information sheet, everything you tell me will remain confidential and if used in any report, will be de-identified. Do you have any questions about this?

Let's start by discussing your experience using iEMR for discharge planning and coordinating post-discharge nutrition care for malnourished older adult patients."

Interview Questions

Current iEMR Use for Discharge Planning

1) Could you first tell me the process you usually follow in iEMR when discharge planning for malnourished older patients?
   - *Prompt: Has using iEMR influenced how you approach discharge planning?*
2) What are your thoughts around using iEMR for discharge planning and coordinating post discharge nutrition care?
   - *Prompts: Is iEMR working well to support this? Why/why not? Is this related to the system or the user or both?*
3) How confident do you feel using iEMR to support discharge planning and coordinating post-discharge nutrition care?
   - *Prompts: What are the main barriers/difficulties you encounter using iEMR for discharge planning and coordinating post-discharge nutrition care? Is there anything that works well/enablers to overcome the barriers you mentioned?*

iEMR Optimisation Opportunities

4) Do you have any thoughts on how the use of iEMR could be further optimised for discharge planning and coordinating post-discharge nutrition care?

5) Do you have any further thoughts on the use of iEMR for discharge planning and coordination of post-discharge nutrition care? What are your thoughts around using iEMR for discharge planning and coordinating post-discharge nutrition care?

For additional context, including study methods and analytic procedures, refer to main manuscript (Sections 2.4 – 3.3). Full participant quotations are provided in Supplementary Material 2.
