# Supplementary material for: ‘Making the System Work’: A Multi-Site Qualitative Study of Dietitians’ Use of iEMR to Support Nutrition Care Transitions for Older Adults with Malnutrition
Source: Healthcare (Basel). 2025 Sep 5;13(17):2227. doi: 10.3390/healthcare13172227 (PMC12428660; doi:10.3390/healthcare13172227)
Supplement: Supplementary file 1 [file healthcare-13-02227-s001.zip › Table S1_Exemplar Quotes.pdf]

Table S1: Extended Exemplary Quotes

Overview

This supplementary material provides extended exemplary quotes supporting each subtheme identified in the qualitative thematic synthesis. These quotes illustrate the rich, contextual insights from participants regarding their use of the integrated electronic medical record (iEMR) system for discharge planning and care coordination.

Extended Exemplary Quotes Table

Table S1. Exemplar Participant Quotes Illustrating Themes and Subthemes Related to Dietitians' Use of iEMR for Discharge Planning and Care Coordination

Participant ID (Role)	Quote	Conceptual Overlap (Cross-Referenced Subtheme)
**Subtheme 1.1: Technical fragmentation, poor interoperability and design limitations compromise care continuity**		
P05 (Dietitian)	"The amount of administration we do for everything…we are double and triple handling everything…Another thing is other professionals identifying dietetic notes...often the doctors set filters that leave out our notes because they are on PowerForms. I have a phone call on everything all the time."	
P11 (Dietitian)	"The issue that I have with EDS summary is if someone's editing it, you can't put anything in and often everyone's editing it in that two hours before discharge and you can't get it in before they finalise it and print it off and it's done. Once the occasion of service has discharged from your hospital, it doesn't really matter anymore."	
P05 (Dietitian)	"I've never heard of any specific instances where a GP has acknowledged receiving it…I've had to call GPs and get them to check that it's been received… the practises have told me that it's been received but it hasn't been opened."	
P12 (Senior Leader)	"There are so many issues with iEMR…I'm not going to get my dietitians to write a chart entry by having to open 20 different things to enter one thing into each area just to make it spit out information at the end…what a waste of time…we're just doing free text."	Also reflects leadership driven documentation workarounds (see Subtheme 2.2)
**Subtheme 1.2: Policy and program eligibility restrictions limit service access and care coordination**		
P02 (Dietitian)	"We try to sort of cheat the system by saying the patient is post-acute so community can pick them up and transition them through My Aged Care that way, but it's problematic…anyone on a level 3 or above package doesn't get seen by our community health services, they need to organise follow-up through their own provider and that often doesn't happen so they get lost to follow-up."	
P07 (Dietitian)	"We would have to make that additional call to their home care provider to see if they have funds for dietitian input...usually for those home care package or NDIS patients, we would try and source the case coordinator or provider to advocate for them and then just hope that it happens."	

**Subtheme 2.1: Trust concerns drive reliance on parallel communication methods**	
P03 (Dietitian)	“I feel like I have to call and make sure someone else is aware of it…I wouldn't solely trust that people are reading my notes and know what's going on, but I do trust that once people know it's there, they do use it.”
P04 (Dietitian)	“There are still certain things where I don't necessarily trust the system…I feel more comfortable sending an email where you get feedback from that person…rather than just doing an EDS or something in the digital space.”
P11 (Dietitian)	“It's almost like you don't trust the processes so you do backups for every process….sometimes when you submit an E-blueslip you're like, ‘Did that go through? Do I do that again?’…I just think we need to have that confidence in our systems.”
**Subtheme 2.2: Documentation and communication workarounds emerge to overcome system functionality limitations**	
P02 (Dietitian)	“I guess the rationale of using our prehistoric forms is that we can just do it at a time that suits us and then it's done…I think the use of iEMR for discharge planning is still frustrating…having multiple systems that we have to utilise…that's a bit clunky and possibly lends itself to human error if something is forgotten or missed.”
P07 (Dietitian)	“When you edit in the form and save it, it looks terrible. We use PowerForms, however, we don't routinely type directly into the designated boxes…we would do it in Word, then paste it into the intervention box…it's just easier, and if we need to amend it, it looks neater for everyone.”
**Subtheme 3.1: Practice variation is shaped by inconsistent cultural expectations and norms**	
P01 (Dietitian)	“For EDS, that is only completed in our rehab population. There's an expectation that all EDS discharge summaries are done, and that's from all Allied Health. The rest of the hospital, there's really no incentive or real expectations to write any of our plans [in the EDS]…there's no accountability or anything like that, no one's checking completion.”
P02 (Dietitian)	“Is it just that we are not embracing change, potentially? I just don't think we've really asked the question to be honest…There's nothing that has prompted us to consider anything different …and when you get used to doing things a certain way, old habits die hard…I think it could be more efficient if we just completed the EDS.”
**Subtheme 3.2: Dietitians’ evolving role and visibility in discharge planning**	
P12 (Senior Leader)	“We constantly get comments from our Director of Allied Health going ‘If you can do anything about discharging a patient, can you get it done?’ and generally that doesn't fall onto dietetics…it's normally pharmacy and physio…and usually they couldn't care less if they'd discharged a patient with or without dietetic input.”
P13 (Senior Leader)	“I think discharge is really hard from a dietetic perspective because when you look at other allied health disciplines, they have more onus of control over discharge because it's functional.”
P14 (Senior Leader)	“It's not something we're looking at now, but the handover is important, and we must have that accountability. If a patient goes somewhere, where are we recording that? We haven't got any clear

	guidelines or policies around what dietitians should be doing…right now, you do what feels like the right thing to do.”	
**Subtheme 3.3: Relational coordination often supersedes structured workflows**		
P06 (Dietitian)	“If I know that they're going into one of our outpatient clinics, I'll have the patient's chart open and just be like, 'Hey, [referring to outpatient colleague], can you come just have a quick look at this patient's chart?’...I think it's a lot less formal [compared to the structured referral processes] when we know they're going to come back.”	
P11 (Dietitian)	“I usually just call the admin staff I know there [referring to a hospital they previously worked at]…ask who's on the ward and they go, ‘oh, this person’, so I send an e-mail handover to that person and CC the hospital.”	
**Subtheme 4.1: Confidence in system value varies by clinical context and user experience**		
P05 (Dietitian)	“Within the same health service, I think it's made discharge planning a smoother process. External to our health service, I feel like my practise is still the same…I will directly contact another dietitian…I'll still do e-mail handover… there's always that direct contact with people so that we know that the patient information is being received.”	Also reflects trust-related adaptations when coordinating externally (see Subtheme 2.1)
P07 (Dietitian)	“Oh, 1000 times better, yes. I think just the live aspect of iEMR…you don't have to go back and read the handwritten paper-based note or wait for all the notes to be written to have a plan. You can refresh and, even if it's a preliminary note you can get the sense of what the plan will be. For time management, it's so much more efficient. I remember you'd wait on the ward for hours just trying to get the chart.”	
P09 (Dietitian)	“I think it has changed the way I practise in the way that I can view things… that snapshot of patient data is more easily visible. We are fortunate in Queensland to have iEMR, it’s the same system and we can see each other's notes.”	
P12 (Senior Leader)	“I honestly think discharge planning and coordination was a lot better in paper-based charts. It feels like with discharge planning in iEMR, there's no efficient way to do it apart from writing a whole separate document… which feels like an extra component of the workload…I don't think it’s easy to use from a discharge planning perspective.”	
P14 (Senior Leader)	“Not for an improvement...not for discharge planning at all...currently, if we've done a handover, it's difficult to find it in iEMR because it can be in a few places. It can be in the EDS or it can be in Viewer or it can be in their notes so....whether iEMR has been a step backwards or forwards, it’s hard to say.”	
**Subtheme 4.2: Analytical capabilities hold potential for transforming service delivery**		
P08 (Dietitian)	“Maybe if there was a way to include an alert somewhere if a patient's due to be discharged that day…that would be really useful... like a little tick box, the team just have to press a tick and it alerts everyone's list they're for discharge.”	
P13 (Senior Leader)	“It's amazing [referring to iEMR dashboards]. What I want, when we get the dietitian dashboard, is for that to be more of our prioritisation tool. We'll still have to use the demand and capacity tool, but that dietitian dashboard will be our data bank for how we	

	prioritise, what the trends look like and how we mobilise to provide care.”
P15 (Senior Leader)	“We're trying to use iView as our way to track outcomes…put in your PES statement…track progress… I think further down the track, people will start looking at outcomes more rather than just activity.”
P16 (Senior Leader)	“We chose to use it [iEMR generated data] for the MST 2s that are automatically referred... because of it, we have now demonstrated clearly over many years since the electronic health record started a 50% increase in our malnutrition assessment and confirmation of malnutrition in the hospital... purely because we utilise the technology.”
**Subtheme 4.3: Integrated and standardised workflows promise more efficient discharge planning**	
P03 (Dietitian)	“Maybe just that consistency of what we should do…whether that's through EDS or not… where it’s just standard across the entire health service. It feels like it's not consistent, so then I feel like I'm having to put things in everywhere to make sure it's not missed.”
P08 (Dietitian)	“If E-blueslip wasn't a separate platform and it was put into iEMR, that would be amazing...If there was maybe a tab on the side and you could just type in and have that information you’ve already documented in iEMR be transferred into the referral, that would make life a lot easier…and maybe if there was a way to include an alert somewhere if a patient is due to be discharged that day.”
P09 (Dietitian)	“Ideally I just want to be able to write my note, say that they need follow up and then it's done and everyone sees that because you've ticked a box and you don't have to do too much other box ticking and coordination… if it was just in that one place where they could look when they needed to…where you can do your last review note plus the plan post hospital discharge and if they need a script or referral…and it's all using the same system.”
P16 (Senior Leader)	“You need far-sighted people to make it standard practice …I do think a lot of these things are almost like an orchestra. We have fabulous musicians all doing their own amazing individual pieces of play, but you need the conductor to bring it together, so it plays harmoniously…you also need the Mozart who envisages the big picture, who can see it coming together as an amazing piece of opera.”

*Some exemplar quotes appear once in full but are reference across multiple subthemes where conceptually relevant.

Summary Contextual Note

Supplementary Table 2 presents participants’ own words, illustrating the challenges, adaptations, cultural influences and optimisation opportunities described in the main manuscript themes and subthemes. These quotes show that the iEMR is experienced not just as a documentation or communication tool, but as part of a broader digital system shaped by technical design, policy constraints, interdisciplinary norms and local workflows.

For additional context, including thematic interpretation and analytic procedures, refer to main manuscript (Sections 2.4-3.3).
