# Supplementary material for: ‘Making the System Work’: A Multi-Site Qualitative Study of Dietitians’ Use of iEMR to Support Nutrition Care Transitions for Older Adults with Malnutrition
Source: Healthcare (Basel). 2025 Sep 5;13(17):2227. doi: 10.3390/healthcare13172227 (PMC12428660; doi:10.3390/healthcare13172227)
Supplement: Supplementary file 1 [file healthcare-13-02227-s001.zip › Table S2_Participant Demographics.pdf]

**Table S2: Participant Characteristics**

## Overview

This supplementary material summarises participant characteristics to provide an overview of the sample. Data are presented in aggregate form only to preserve confidentiality, as greater granularity (e.g., site-level detail) could risk re-identification. The sample included a mix of frontline clinical dietitians and senior dietetics leaders across multiple Queensland public hospitals, with variation in experience, tenure, caseloads and prior experience in paper-based systems.

## Participant Characteristics Table

**Table S2.** Summary of Participant Characteristics (n = 16)

| Characteristic | n (%) or Median (IQR) | Notes |
|---|---|---|
| Role | 11 (69%) frontline clinical dietitians; 5 (31%) senior leaders | Senior leaders include directors, team leaders and research dietitians (grouped for anonymity). |
| Gender (self-reported) | 14 females; 2 males | Gender data reflects self-identification where provided. |
| Hospital sites represented | 10 sites across 6 Queensland HHSs | Aggregate only; site-level detail not reported for confidentiality |
| Years of clinical experience | Median 6.0 years (IQR 3.0 – 15.8) | Nine participants had ≤ 6 years of experience. |
| Tenure at current site | Median 3.0 years (IQR 1.3 – 11.5) | - |
| Previous experience in paper-based systems | 7 (44%) | Remaining participants had only practised in digital environments |
| Caseload types | Mix of medical, surgical, or combined; 4 also covered outpatient or telehealth services | Broad categories only to preserve anonymity. |
| Interview duration | Median 39 minutes (range 16-56; IQR 34-44) | - |

## Summary Contextual Note

This sample reflected a diverse cross-section of hospital-based dietitians working within a common digital health infrastructure. These contextual and demographic characteristics provide important background for interpreting participants' insights into iEMR use, local adaptations and nutrition care discharge practices across sites.
